# Are Fibrous Cortical Defects (FCDs) and Non-Ossifying Fibromas (NOFs) Only Radiological Findings? The Relationship between Radiological/Clinical Findings and Physical Activity in Children and Adolescents: A Cross-Sectional Study

**DOI:** 10.3390/jcm13195751

**Published:** 2024-09-27

**Authors:** Erhan Berk, Rabia Aydogan Baykara

**Affiliations:** 1 Department of Pediatrics, Malatya Turgut Ozal University, 44210 Malatya, Turkey; erhan.berk@ozal.edu.tr; 2 Department of Physical Medicine and Rehabilitation, Malatya Turgut Ozal University, 44210 Malatya, Turkey

**Keywords:** fibrous cortical defect (FCD), non-ossifying fibroma (NOF), clinical findings, physical activity, children, adolescents, 21-numbered circle activity scale, IPAQ, 21-numbered circle VAS

## Abstract

**Background:** Fibrous cortical defect (FCD) and non-ossifying fibroma (NOF) are incidentally recognised and benign developmental lesions. The objective of this study was to ascertain the clinical manifestations and symptoms of FCDs/NOFs in children and adolescent patients, to characterise the lesions radiologically using X-ray and MRI techniques, and to determine the relationship between physical activity and the condition. **Methods:** The study included patients under the age of 18 with radiological lesions on their extremities. The lesions were classified as FCD or NOF in accordance with the distinctive imaging features. For each lesion, the bone involved, the site involved, the size of the lesion, and the type of lesion (according to the Ritschl classification) were recorded. In the anamnesis, the patient’s presenting complaint, the character of the pain, if any, and the level of activity were investigated. Pain was quantified using the visual analogue scale (VAS) and the 21-Numbered Circle VAS (21-NCVAS). The 21-Numbered Circle Activity Scale (21-NCAS) and the International Physical Activity Questionnaire (IPAQ) were employed for the assessment of physical activity. **Results:** The study included 34 lesions in 28 children (14 girls/14 boys). There was no difference in age between girls and boys (*p* = 0.45). According to Ritschl’s classification, 18 (52.9%) lesions were stage A, 9 (26.5%) were stage B, and 7 (20.6%) were stage C. The lesion size increased with increasing Ritschl stage (*p* < 0.02). The main presenting complaint was pain (n = 13, 49.9%). In 21.4% of the children (n = 6), lesions were detected incidentally on radiographs. According to IPAQ, 39.3% of the children were physically inactive. There was a significant negative correlation between 21-NCAS and Ritschl stage (r = −0.51, *p* < 0.05). Activity decreased as the Ritschl stage increased. There was a significant negative correlation between 21-NCAS and VAS (r = −0.69, *p* < 0.05). **Conclusions:** Spontaneous pain was observed in 49.9% of patients diagnosed with FCD/NOF. No correlation was identified between lesion size and the presence or severity of pain. As the severity of pain and Ritschl stage increased, there was a corresponding decrease in physical activity.

## 1. Introduction

Fibrous cortical defect (FCD) and non-ossifying fibroma (NOF) are benign lesions that occur with the presence of osteoclast-like multinucleated giant cells accompanied by fibroblast proliferation on pathological examination [1,2]. Although their histopathology is similar, the terminology differs such that lesions smaller than 2 cm and remains confined to the bone cortex, are named as “fibrous cortical defect”, whereas bigger lesions with variable endo medullary extension are named as “non-ossifying fibroma” [2,3,4,5]. Accordingly, the two aforementioned conditions were consolidated into a single category, designated as FCD/NOF, for the purposes of this study.

These lesions are regarded as developmental variations, manifesting predominantly during the first and second decades of life [2,6]. These lesions represent the most common benign lesions of the skeletal system, with an estimated prevalence of 30–40% in skeletally immature children [5,6,7,8]. A study of the Japanese population demonstrated a prevalence of NOF of 2.3% and FCD of 7.0%. NOF was more prevalent in boys, while FCD was more prevalent in girls [7]. Genetic predisposition for NOF was confirmed by DNA sequencing and MAP kinase signalling [2]. Additionally, Nelson et al. reported chromosomal translocation bands in a patient diagnosed with NOF [9].

As with other benign bone tumours, FCD and NOF are initially diagnosed through clinical evaluation and other conventional imaging modalities, such as X-ray [4,10]. If necessary, advanced diagnostic modalities, such as computed tomography and magnetic resonance imaging are employed to supplement the initial assessment [3,4]. Following preliminary examination, staging and prognostic evaluations should be conducted according to histological type and anatomical localisation. The Enneking staging system is the most widely accepted system for benign bone tumours. Staging is important in determining the type of surgical treatment. The size, depth, growth rate, anatomical location, and histological features of the lesion can guide in determining the behaviour of the lesion and predicting the prognosis [11]. Characteristic imaging findings of the lesions include being eccentric within the bone and adjacent to the cortex, moving close to the physis or towards the metaphysis during growth, and having a sclerotic margin [6]. The long axis of the lesions is parallel to the long axis of the bone, while lesions are most commonly localized around the knee, including the distal femur and proximal medial tibia [6,12], and very rarely in the mandible [13].

When evaluated clinically, FCD is usually asymptomatic and is detected incidentally on an X-ray taken for another reason, and does not require any intervention [3,7]. NOF is rarer and more eccentric compared to FCD, while these lesions usually disappear spontaneously with age [3]. Sometimes the lesions may be palpably swollen, compress adjacent muscle or neurovascular structures, and expand the cortex, causing damage to adjacent bone [2,3]. Symptoms are generally described as pain, swelling and tenderness, and sometimes fracture, and treatment is usually conservative, but if trauma is present and causes symptoms and bone weakening, prophylactic treatment may be required [13,14,15]. Fractures are often seen in the lower extremity, at the distal end of the tibia. In addition, if the lesion covers more than 50% of the bone in 2 projections and is longer than 33 mm, the risk of bone fracture is considered high, and surgical bone grafting and local intralesional curettage are sufficient in its treatment [5,13,14,16]. Nevertheless, a more recent series of studies demonstrated that 59% of NOF cases exceeded the aforementioned threshold measurements without fracture [16]. 

Pain and limitation of movement can negatively affect growth and development in children and adolescents, while pain, fractures, and other symptoms caused by FCD/NOF might hinder activity and increase physical inactivity which might negatively affect both physical and psychosocial development in children and young adolescents [17]. According to WHO 2020 guidelines, physical activity is necessary for healthy growth and development, mental health, social skills development, attention concentration, and learning ability in children and adolescents [18]. Thus, symptoms of FCD/NOF might reduce the level of physical activity required for healthy development in children and increase physical inactivity.

The aims of this study are to determine the symptoms that occur in children diagnosed with FCD/NOF, to reveal the characteristics of the X-ray and MRI images of the lesions, and to evaluate the relationship of physical activity.

## 2. Material and Methods

### 2.1. Patient Selection

The sample was selected from patients with imaging records in the system for the previous five years and who were under the age of 18 at the time of diagnosis. The patients were identified by searching the diagnosis records in the hospital database for the keywords “fibrous cortical defect” and “non-ossifying fibroma”. The same search was conducted in the radiology reports. The patients were contacted by telephone. Those who agreed to participate were invited to the hospital where they signed a consent form. Demographic data of patients such as age, gender, education, and other variables such as initial complaint, complaint duration, comorbidity, family history of rheumatological disease, and screen time were recorded. The reason for admission to the hospital, trauma history, and extensive anamnesis were collected. The same physician (RAB) performed an examination, and the patient’s activity levels were assessed. The questionnaires were administered to the patients individually, but parents were permitted to contribute to the process.

The imaging findings of the children’s lower extremities, as well as the standard anteroposterior and lateral X-ray images and MRI images, were obtained from the hospital records as previously mentioned and within the specified time interval. Patients were included in the study according to the criteria: (1) patients aged 10–18 years, and (2) patients who underwent standard anteroposterior and lateral view radiography and/or (3) MRI. 

Exclusion criteria: (1) patients with diagnosed metabolic and endocrine diseases, other diseases affecting bone metabolism, rheumatological diseases, neurological diseases, a history of fractures and operations other than FCD/NOF, and (2) patients whose radiological imaging quality is not sufficient for the study were excluded from the research.

### 2.2. Radiological Evaluation of FCD/NOF

For all lesions, the intraosseous involvement and size of the lesion were evaluated and lesions were classified according to the Ritschl classification: Stage A: Small, oval to slightly polycyclic in shape, without a sclerotic border, in the cortex near the epiphyseal end plate. Stage B: Lesions at a variable distance from the epiphysis, polycyclic in shape, with thin but clear sclerotic borders and a thin cortex occasionally protruding above the surface in an hourglass shape without periosteal reaction. Stage C: Lesions with partial sclerosis. Stage D: Lesions with complete sclerosis [19]. The Ritschl classification is based on the clinical course of the healing process and these stages are proportional to the age of the patient. Stage B lesions have an increased risk of fracture, therefore follow-up until stage C is reached is recommended [6].

#### 2.2.1. Pain Assessment

For pain assessment, both 10 cm horizontal line visual analog scales (VAS) [20] and 21-numbered circle visual analog scales (21-VAS) were used, described elsewhere in detail [20,21]. The Likert-type scale, which increases in 0.5 cm intervals, has an unhappy face emoji on the 0 side and a smiling face emoji on the side where 10 is placed. This scale is more useful in children as it is completed faster and shows remission better than the standard 10 cm VAS [20,21].

The main complaints were categorized as: (1) Pain, (a) Pain with movement, (b) Pain at rest, (c) Night Pain, (d) Pain while running, (2) Swelling and Tenderness, (3) Chronic Pain <3 month, (4) Difficulty in walking, (5) Instability in weight bearing, (6) Fracture. Morning stiffness, shifting pain in the hip, fatigue, as well as screen time were questioned in the anamnesis. In the examination, swelling, increase in body temperature, and tenderness were evaluated.

#### 2.2.2. Physical Activity Scale

21-numbered circle activity scale (21-NCAS): There is “no activity” on the 0 side of the Likert-type scale, increasing at 0.5 cm intervals, and “maximum activity” on the 10 side. High scores indicate that the activity can be performed easily.

International Physical Activity Questionnaire (IPAQ): The last seven days of activity were evaluated with the questionnaire. Walking and activity are evaluated as duration (minutes) and frequency (days) and the total score is classified as “very active”, “moderately active” or “inactive”. Sitting: 1.5 MET, walking: 3.3 MET, moderate physical activity: 4.0 MET, vigorous physical activity: 8.0 MET. For example, for a person who walks for 7 days and 30 min, the MET-min/week score is calculated as: 3.3 × 7 × 30 = 693 MET-min/week [22].

Statistical analyses: The data analysis was conducted by using the IBM SPSS 25 statistical software package, and the data’s conformity to normal distribution was tested by using the Kolmogorov–Smirnov test. Descriptive statistics were presented as mean ± SD, median (range), and n (%). The independent Student’s *t*-test and chi-square test were used to compare the two groups, while the one-way ANOVA test was used to compare more than 2 groups, and the Tukey *t*-test was used for post hoc analysis at a significance level of *p* < 0.05. The study also encompassed correlation analyses using the Pearson correlation method. For the evaluation of categorical data, cross-tabulation was used in conjunction with the chi-square test.

Before participating in the study, written informed consent was obtained from all participants. The study was approved by the Local Clinical Research Ethics Committee, which previously worked according to the evaluation procedure by taking into account the Council of Europe guidelines and the Declaration of Helsinki warnings, with the decision number E-30785963-020-222774/2024/43.

## 3. Results

A total of 34 lesions (three children had more than one lesion) in 28 children (14 girls/14 boys) with a mean age of 14.5 ± 1.8 years for girls, and 15.14 ± 2.5 years for boys, were included in the study (*p* = 0.45).

Clinical Evaluation: The patients applied to the hospital presented: pain (n = 13, 49.9%), instability in weight-bearing (n = 6, 21.4%), pain with movement (n = 10, 35.7%), pain while running (n = 4, 14.3%), night pain (n = 6, 21.4%), pain at rest (n = 7, 25%), morning stiffness (n = 4, 14.3%), chronic pain (lasting more than 3 months) (n = 8, 28.6%), fatigue (n = 4, 14.3%), tenderness on examination (n =16, 57.1%). The lesions of 21.4% (n = 6) of 28 children were detected incidentally on radiographs, while the complaints of boys and girls did not differ when they came to the hospital (*p* > 0.05), but pain at rest was more common in boys compared to girls when symptoms were questioned (*p* < 0.05) (Table 1).

According to IPAQ, 39.3% of the children were inactive, 39.3% moderately active and 21.4% very active, while no statistically significant difference was observed in physical activity between boys and girls (*p* > 0.05). The IPAQ MET mean was calculated as 1455 ± 1215 presented in detail in Table 2.

According to the results for correlation analysis, there was a negative significant relationship between the 21-NCAS score and the scores of VAS, 21-VASand the Ritschl stage, (r = −0.69, r = −0.56, r = −0.51), respectively (*p* < 0.05). There was no difference between boys and girls in terms of the Ritschl stage (*p* > 0.05). There was no difference between the time spent in front of the screen and parameters such as exercise scales (activity scale number 21 and IPAQ) and the Ritschl stage (Table 3).

As Ritschl stage and pain level increase, physical activity level decreases. However, no correlation was identified between pain level and Ritschl stage.

**Figure 1:**
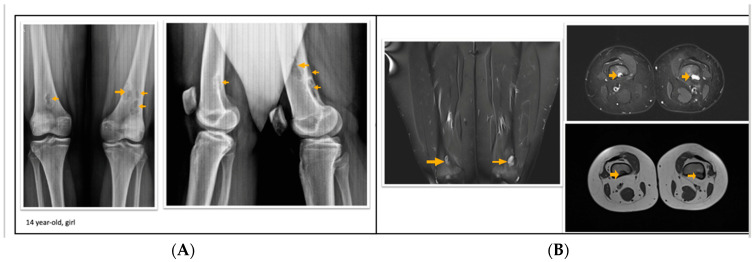
A 14-year-old girl with multiple fibrous cortical defects around the knee. (**A**) Direct X-ray of the knee in anteroposterior and lateral aspects show eccentrically located posteromedial and posterolateral lytic lesions with a sclerotic rim consistent with fibrous cortical defect. (**B**) Coronal and axial proton density and axial T1 weighted MR sections show lesions in posterior and posteromedial aspects of both femurs consistent with fibrous cortical defects. These lesions were classified as stage A according to Ritschl’s classification. Clinically, IPAQ (International Physical Activity Questionnaire): Moderately active, Screen Time: 3 h, 21-Numbered Circle VAS (21-VAS): 4.5, VAS (Visual analogue scale): 4, 21-Numbered Circle Activity Scale (21-NCAS): 6.

**Figure 2:**
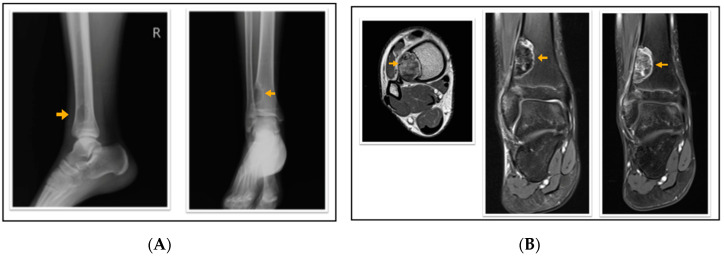
A 16-year-old patient with a fibrous cortical defect in the ankle. (**A**) Lateral and anteroposterior direct X-ray of a 16-year-old patient shows a lytic lesion with a sclerotic rim in the distal medial metaphysis of the tibia consistent with FCD. (**B**) Axial T1 weighted, coronal proton density, and coronal contrast-enhanced fat-saturated T1 weighted magnetic resonance images show sclerotic, non-contrast enhancing areas within the lesion. This lesion was classified as stage C according to Ritschl’s classification. Clinically, IPAQ (International Physical Activity Questionnaire):Inactive, Screen Time: 4 h, 21-Numbered Circle VAS (21-VAS): 3, VAS (Visual analogue scale): 3, 21-Numbered Circle Activity Scale (21-NCAS): 5.

**Figure 3:**
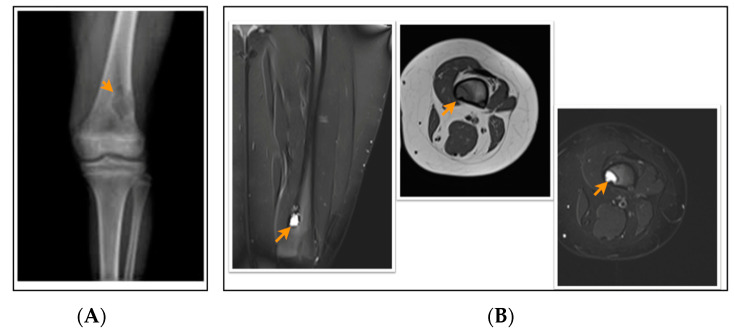
A 12-year-old patient with a fibrous cortical defect around the knee. (**A**) Anteroposterior direct X-ray of a 12-year-old patient shows a lytic lesion with a sclerotic rim in the distal posteromedial metaphysis of the femur consistent with FCD. (**B**) Coronal and axial proton density and axial T1-weighted MR sections of the posteromedial left femur show a lesion consistent with a fibrous cortical defect. This lesion was classified as stage B according to Ritschl’s classification, because of lobulated contour. Clinically, IPAQ (International Physical Activity Questionnaire): Inactive, Screen Time: 5 h, 21-Numbered Circle VAS (21-VAS): 4, VAS (Visual analogue scale): 4, 21-Numbered Circle Activity Scale (21-NCAS): 4.

According to the radiological evaluation results, 34 lesions had X-ray scans available in the archive of the hospital. One of the patients had 3 lesions, and 2 patients had 2 lesions, while the rest had only 1 lesion, summarized as 34 lesions in 28 patients. All lesions were eccentrically located within the metaphysis of a long bone such that 22 lesions were in the femur and 12 lesions were in the tibia, 18 of the lesions were on the left side (Figure 1, Figure 2 and Figure 3). Only 4 lesions were examined with contrast-enhanced MRI; from these 3 had peripheral and only 1 had heterogeneous contrast enhancement after intravenous contrast media injection (Figure 1, Figure 2 and Figure 3).

The mean size of the lesions was 3.68 ± 4.75. (0.12–21.74) cm^3^. According to Ritschl’s classification, 18 (52.9%) lesions were stage A, 9 (26.5%) lesions were stage B, 7 (20.6%) lesions were stage C. Lesion size in stage C was higher compared to stage A and stage B, while lesion size increases as the Ritschl stage increased from stage A to stage B and to stage C continuously (*p* < 0.02). All groups showed a statistically significant difference when compared to each other (*p* < 0.05)and as the lesion size also increased (*p* < 0.02). The age variable did not show any significant difference among groups of the Ritschl stage (*p* > 0.05) (Table 4).

## 4. Discussion

This study is the first to investigate the relationship between the radiological characteristics and clinical evaluation of FCD/NOF and the physical activity level in children. In our study, we observed that FCD/NOF lesions occurred incidentally in 21.4% of patients. Despite the assertion in the existing literature that lesions are predominantly identified on radiographs obtained for reasons unrelated to the detection of such lesions, our study revealed that the most frequently reported symptom was pain, accounting for 49.9% of cases. Similarly, in a prevalence study, half of the patients with FCD had spontaneous pain complaints [5,7]. 

When complaints were compared between boys and girls, no significant difference was found between them, but pain at rest was seen to be more common in boys. The physical activity levels of children and adolescents were classified as either very inactive or moderately active, a finding that was consistent with that of previous published literature [23]. Parallel findings were found in a large-scale study conducted by R. Guthold et al., covering 1.6 million children and young adolescents, stating that 81% of the students aged between 11 and 18 years (boys: 77.6%/girls: 84.7%) were not physically active [20,21,23,24]. A further noteworthy finding of the study was that, while the physical activity levels of boys and girls with FCD/NOF were similar, there was a decline in physical activity as the Ritschl stage increased. Moreover, physical activity was associated negatively with pain scores. Thus, pain is considered as a condition that limits physical activity. Similar to our findings, a study evaluating pain and physical activity bidirectionally in adolescents found that the experience of pain limits physical activity on a daily basis. In a study comparing adolescents with chronic pain and their healthy peers, physical activity was measured objectively via actigraphy, and it was found that those with pain had lower levels of physical activity as well as decreased physical functionality [25]. However, in adolescents with chronic pain, the pain intensity was lower at the end of the day in those with higher levels of physical activity [26]. The findings of our study showed that there is no relationship between the Ritschl stage and the pain scores. In our study, lesion size increased with increasing Ritschl stage. Based on this information, it can be considered that an increased lesion size is not associated with pain, as Emori et al. reported that lesion size is not associated with spontaneous pain [7].

On the other hand, the use of technology may cause a decrease in physical activity because it hinders children’s daily activities. In previous studies, physical activity was found to be low in children who spent along time using technology [27,28]. In our study, the majority of children with FCD/NOF had more than 2 h of screen time in both genders, but screen time was not associated with physical activity level. Parallel results were obtained in the study conducted by Karaca et al., which showed that screen time was not correlated with physical activity in children without any disease [29], and Nilsson et al. who reported that increasing exercise time was not correlated to shortening TV watching [30]. An international study conducted by Melkevik et al. showed that screen time is negatively correlated to physical activity in countries where the average physical activity level is high. However, across geographic regions and genders with generally lower levels of physical activity, it has been shown that there is no consistent association between levels of screen-based sedentary behaviour of more than 2 h per day and physical activity [31].

According to our radiological findings, stage A lesions were the most detected lesion classified according to Ritschl’s classification, which was consistent with the findings of the study conducted by Emori et al. [7]. Additionally, our study showed that size of FCD or NOF increases with the increasing stage according to Ritschl’s classification, while Herget et al. also reported that the size of the lesions increased with the increase in the stage of the lesions [6]. In contrast to the study of Herget et al. who reported an increase in age with increasing Ritschl stage, we did not find any significant difference between Ritschl stages according to age. Blaz et al. also agreed that the average age of patients increased with increasing Ritschl stage [4,6], both studies included adults in addition to children in their study sample. Thus, different results in our study might be due to the younger age group of patients and using different imaging methods for evaluation.

In our study, 9 patients were in stage B, of which one had a pathological fracture, which is consistent with the literature stating that patients with stage B lesions have an increased risk of suffering a pathological fracture. Similarly, Herget et al. reported pathological fractures in 6 out of 87 patients [6], while Emori et al. reported that fractures were detected in 2.1% of patients with Ritschl classification stage B [7]. Moreover, 3 children had more than one lesion in our study which is also consistent with literature stating that 5–8% of patients have multiple lesions [6,7,32].

This study has several limitations that have to be stated. Firstly, the patient’s exercise barriers, which might affect the physical activity level, have not been questioned in detail. Moreover, some physical activity parameters such as intensity and type of exercise (i.e., aerobic and muscle and bone strengthening activities) have not been identified. Secondly, children were also not evaluated regarding sleep duration and psychological impact. In addition, patient selection was based on imaging findings, which is one of the important limitations. No biopsy or pathological diagnosis was performed in this study. Finally, although we classified lesions according to Ritschl’s classification, a longitudinal study that follows patients over the long term could show the progression of X-ray and MRI findings over time. The Ritschl staging system is based on the clinical course of the healing process, as the stage increases, the lesion is expected to mature and then heal. The progression of the Ritschl stage with increasing child age has been documented [6]. The absence of Ritschl stage D in our sample (because stage D cases over 18 years of age were not included in the study) and the small sample size can be considered as other limitations.

## 5. Conclusions

The first outcome of the research is that this study revealed physical activity, radiological imaging and clinical status in children diagnosed with FCD/NOF. The clinical background of this pathology, known as “touch-free lesion” in the literature, was evaluated.

Secondly, the relationship between pain and other symptoms and physical activity was examined, and attention was drawn to physical activity affecting the growth and development of children.

It was determined that pain was the main complaint in half of the patients and was responsible for restricting mobility and thereby decreasing physical activity in patients. In addition, as the Ritschl stage increased, physical activity decreased. Therefore, it is recommended to inform the child and the family about the decrease in physical activity and to provide support from experts when necessary.

## Figures and Tables

**Table 1:** Comparison of patients according to pain and clinical evaluation.

		Gender		Total	*p*
		Girls (n (%))	Boys (n (%))		
Complaints					
	Pain	5 (35.7)	8 (57.1)	13 (49.9)	0.25
	Night Pain	2 (14.3)	4 (28.6)	6 (21.4)	0.35
	Pain at Rest	1 (7.1)	6 (42.9)	7 (25.0)	0.02 *
	Pain with Movement	2 (14.3)	1 (7.1)	3 (10.7)	0.35
	Pain while Running	2 (14.3)	2 (14.3)	4 (14.3)	0.35
	Duration of Chronic Pain <3 months	2 (14.3)	6 (42.9)	8 (28.6)	0.09
	Difficulty in Walking	1 (7.1)	1 (7.1)	2 (7.1)	0.35
	Instability in Weight-bearing	3 (21.4)	3 (21.4)	6 (21.4)	0.35
	Morning Stiffness	2 (14.3)	2 (14.3)	4 (14.3)	1
	Fatigue	2 (14.3)	2 (14.3)	4 (14.3)	1
	Fracture	0.0	1 (7.1)	1 (3.6)	0.35
No complaint, coincidence		5 (35.7)	1 (7.1)	6 (21.4)	0.35
Sensitivity in examination		6 (42.9)	10 (71.4)	16 (57.1)	0.12
Screen Time #	>2 h	3 (21.4)	2 (14.3)	5 (17.9)	0.62
	<2 h	11 (78.6)	12 (85.7)	23 (82.1)	

* Pearson chi-square was considered significant at *p* < 0.05. # Screen Time is divided into two groups: more than 2 h or less than 2 h, m: Month.

**Table 2:** Comparison of physical activity level according to gender measured by IPAQ scale.

	Gender			
IPAQ	Girls (n (%))	Boys (n (%))	Total (n (%))	*p*
Inactive	4 (28.6)	7 (50)	11 (39.3)	0.19
Moderately active	9 (64.3)	2 (14.3)	11 (39.3)	
Very active	1 (7.1)	5 (35.7)	6 (21.4)	
Total	14 (100)	14 (100)	28 (100)	

Pearson chi-square was considered significant at *p* < 0.05, IPAQ: International Physical Activity Questionnaire.

**Table 3:** Correlation of pain scales, physical activity scales, screen time, and Ritschl classification.

		IPAQ	21-NCAS	VAS	21-VAS	SET	RITSCHL
IPAQ	r	1	−0.001	−0.094	−0.024	−0.186	−0.138
	*p*		0.996	0.633	0.902	0.343	0.483
21-NCAS	r		1	−0.697 *	−0.563 *	0.039	−0.514 *
	*p*			<0.001	0.002	0.844	0.005
VAS	r			1	0.867 *	0.087	0.359
	*p*				<0.001	0.661	0.061
21-VAS	r				1	0.105	0.359
	*p*					0.593	0.061
SET	r					1	0.201
	*p*						0.305
RITSCHL	r						1

* Correlation is significant at the 0.05 level (2-tailed). IPAQ: International Physical Activity Questionnaire, SET: Screen Time, 21-Numbered Circle VAS (21-VAS), VAS: Visual analog scale, 21-Numbered Circle Activity Scale (21-NCAS), RITSCHL:Ritschl’s classification stage.

**Table 4:** Size of lesions and age according to the Ritschl classification.

	Number of Lesions	Size (cm^3^) Mean (±SD) Min–Max	*p* ^#^	Mean(±SD) Min–Max	*p* ^&^
Stage A	18	1.63 (±0.30) 0.32–3.4	0.022 ^1^	14.2 (±2.17) 10–17	0.13
Stage B	9	5.96 (±1.67) 0.39–15.10	0.022 ^2^	14.6 (±1.6) 11–16	
Stage C	7	6.22 (±7.56) 0.35–21.74	0.026 ^3^	15.8 (±1.6) 13–18	
Total	34	3.68 (±4.75) 0.12–21.74		18	

*p*^#^ value is the result of comparison of three groups (Stage A, B, C), post hoc results for comparison; ^1^: Stage B, C, ^2^: Stage A–C, ^3^: Stage A, B, *p*^&^ value is the result of comparison of three groups (Stage A, B, C). A one-way ANOVA was used.

## Data Availability

The datasets are not publicly available but are available from the corresponding author upon reasonable request.

## References

[1] Schajowicz F. (2012). Histological Typing of Bone Tumours.

[2] Baumhoer D., Rogozhin D. (2020). Non-ossifying fibroma. Soft Tissue and Bone Tumours.

[3] Mankin H.J., Trahan C.A., Fondren G., Mankin C.J. (2009). Non-ossifying fibroma, fibrous cortical defect and Jaffe—Campanacci syndrome: A biologic and clinical review. Musculoskelet. Surg..

[4] Błaż M., Palczewski P., Swiątkowski J., Gołębiowski M. (2011). Cortical fibrous defects and non-ossifying fibromas in children and young adults: The analysis of radiological features in 28 cases and a review of literature. Pol. J. Radiol..

[5] Betsy M., Kupersmith L.M., Springfield D.S. (2004). Metaphyseal fibrous defects. J. Am. Acad. Orthop. Surg..

[6] Herget G.W., Mauer D., Krauß T., El Tayeh A., Uhl M., Südkamp N.P., Hauschild O. (2016). Non-ossifying fibroma: Natural history with an emphasis on a stage-related growth, fracture risk and the need for follow-up. BMC Musculoskelet. Disord..

[7] Emori M., Tsuchie H., Teramoto A., Shimizu J., Mizushima E., Murahashi Y., Nagasawa H., Miyakoshi N., Yamashita T. (2022). Non-ossifying fibromas and fibrous cortical defects around the knee—An epidemiologic survey in a Japanese pediatric population. BMC Musculoskelet. Disord..

[8] Sontag L.W., Pyle S.I. (1941). The appearance and nature of cyst-like areas in the distal femoral metaphyses of children. Am. J. Roentgenol..

[9] Nelson M., Perry D., Ginsburg G., Sanger W.G., Neff J.R., Bridge J.A. (2003). Translocation (1;4)(p31;q34) in nonossifying fibroma. Cancer Genet. Cytogenet..

[10] Rammanohar J., Zhang C., Thahir A., Krkovic M. (2021). Imaging of non-ossifying fibromas: A case series. Cureus.

[11] Enneking W.F., Spanier S.S., Goodman M.A. (1980). A system for the surgical staging of musculoskeletal sarcoma. Clin. Orthop. Relat. Res. (1976–2007).

[12] Sanatkumar S., Rajagopalan N., Mallikarjunaswamy B., Srinivasalu S., Sudhir N., Usha K. (2005). Benign fibrous histiocytoma of the distal radius with congenital dislocation of the radial head: A case report. J. Orthop. Surg..

[13] Bowers L.M., Cohen D.M., Bhattacharyya I., Pettigrew J.C., Stavropoulos M.F. (2013). The non-ossifying fibroma: A case report and review of the literature. Head. Neck Pathol..

[14] Hernanz López P., Moreno Cano P., Bello González C. (2018). Non-ossifying fibroma. Semergen.

[15] Rogozhin D.V., Konovalov D.M., Kozlov A.S., Talalaev A.G., Ektova A.P. (2016). Non-ossifying fibroma (metaphyseal fibrous defect). Arkh Patol..

[16] Easley M.E., Kneisl J.S. (1997). Pathologic fractures through nonossifying fibromas: Is prophylactic treatment warranted?. J. Pediatr. Orthop..

[17] Sampasa-Kanyinga H., Colman I., Goldfield G.S., Janssen I., Wang J., Podinic I., Tremblay M.S., Saunders T.J., Sampson M., Chaput J.P. (2020). Combinations of physical activity, sedentary time, and sleep duration and their associations with depressive symptoms and other mental health problems in children and adolescents: A systematic review. Int. J. Behav. Nutr. Phys. Act..

[18] Chaput J.P., Willumsen J., Bull F., Chou R., Ekelund U., Firth J., Jago R., Ortega F.B., Katzmarzyk P.T. (2020). 2020 WHO guidelines on physical activity and sedentary behaviour for children and adolescents aged 5–17 years: Summary of the evidence. Int. J. Behav. Nutr. Phys. Act..

[19] Ritschl P., Karnel F., Hajek P. (1988). Fibrous metaphyseal defects—Determination of their origin and natural history using a radiomorphological study. Skelet. Radiol..

[20] Filocamo G., Davì S., Pistorio A., Bertamino M., Ruperto N., Lattanzi B., Consolaro A., Magni-Manzoni S., Galasso R., Varnier G.C. (2010). Evaluation of 21-numbered circle and 10-centimeter horizontal line visual analog scales for physician and parent subjective ratings in juvenile idiopathic arthritis. J. Rheumatol..

[21] Pincus T., Bergman M., Sokka T., Roth J., Swearingen C., Yazici Y. (2008). Visual analog scales in formats other than a 10 centimeter horizontal line to assess pain and other clinical data. J. Rheumatol..

[22] Savcı S., Öztürk M., Arıkan H., İnal İnce D., Tokgözoğlu L. (2006). Physical activity levels of university students. Arch. Turk. Soc. Cardiol..

[23] Guthold R., Stevens G.A., Riley L.M., Bull F.C. (2020). Global trends in insufficient physical activity among adolescents: A pooled analysis of 298 population-based surveys with 1·6 million participants. Lancet Child. Adolesc. Health.

[24] Tremblay M.S., Carson V., Chaput J.P., Connor Gorber S., Dinh T., Duggan M., Faulkner G., Gray C.E., Gruber R., Janson K. (2016). Canadian 24-Hour Movement Guidelines for Children and Youth: An Integration of Physical Activity, Sedentary Behaviour, and Sleep. Appl. Physiol. Nutr. Metab..

[25] Wilson A.C., Palermo T.M. (2012). Physical activity and function in adolescents with chronic pain: A controlled study using actigraphy. J. Pain..

[26] Rabbitts J.A., Holley A.L., Karlson C.W., Palermo T.M. (2014). Bidirectional associations between pain and physical activity in adolescents. Clin. J. Pain..

[27] Alotaibi T., Almuhanna R., Alhassan J., Alqadhib E., Mortada E., Alwhaibi R. (2020). The Relationship between Technology Use and Physical Activity among Typically-Developing Children. Healthcare.

[28] McDougall J., Duncan M.J. (2008). Children, video games and physical activity: An exploratory study. Int. J. Disabil. Human. Dev..

[29] Karaca A., Caglar E., Bilgili N., Ayaz S. (2011). Screen time of adolescents in an economically developing country: The case of Turkey. Ann. Hum. Biol..

[30] Nilsson A., Andersen L.B., Ommundsen Y., Froberg K., Sardinha L.B., Piehl-Aulin K., Ekelund U. (2009). Correlates of objectively assessed physical activity and sedentary time in children: A cross-sectional study (The European Youth Heart Study). BMC Public. Health.

[31] Melkevik O., Torsheim T., Iannotti R.J., Wold B. (2010). Is spending time in screen-based sedentary behaviors associated with less physical activity: A cross national investigation. Int. J. Behav. Nutr. Phys. Act..

[32] Moser R.P., Sweet D.E., Haseman D.B., Madewell J.E. (1987). Multiple skeletal fibroxanthomas: Radiologic-pathologic correlation of 72 cases. Skelet. Radiol..

